# Supplementary material for: Effect of Poria cocos Terpenes: Verifying Modes of Action Using Molecular Docking, Drug-Induced Transcriptomes, and Diffusion Network Analyses
Source: Int J Mol Sci. 2024 Apr 24;25(9):4636. doi: 10.3390/ijms25094636 (PMC11083729; doi:10.3390/ijms25094636)
Supplement: Supplementary file 1 [file ijms-25-04636-s001.zip › Supplementary Figures S1-S3.pdf]

	Eburicoic acid (Lanostane triterpenes) -9.704 kcal/mol	Poricoic acid A (seco-Lanostane triterpenes) -8.563 kcal/mol	7-Oxodehydroabietic acid (Tricyclic diterpenes) -7.817 kcal/mol
3D-Ribbons			
2D-diagrams			
Molecular Surface			

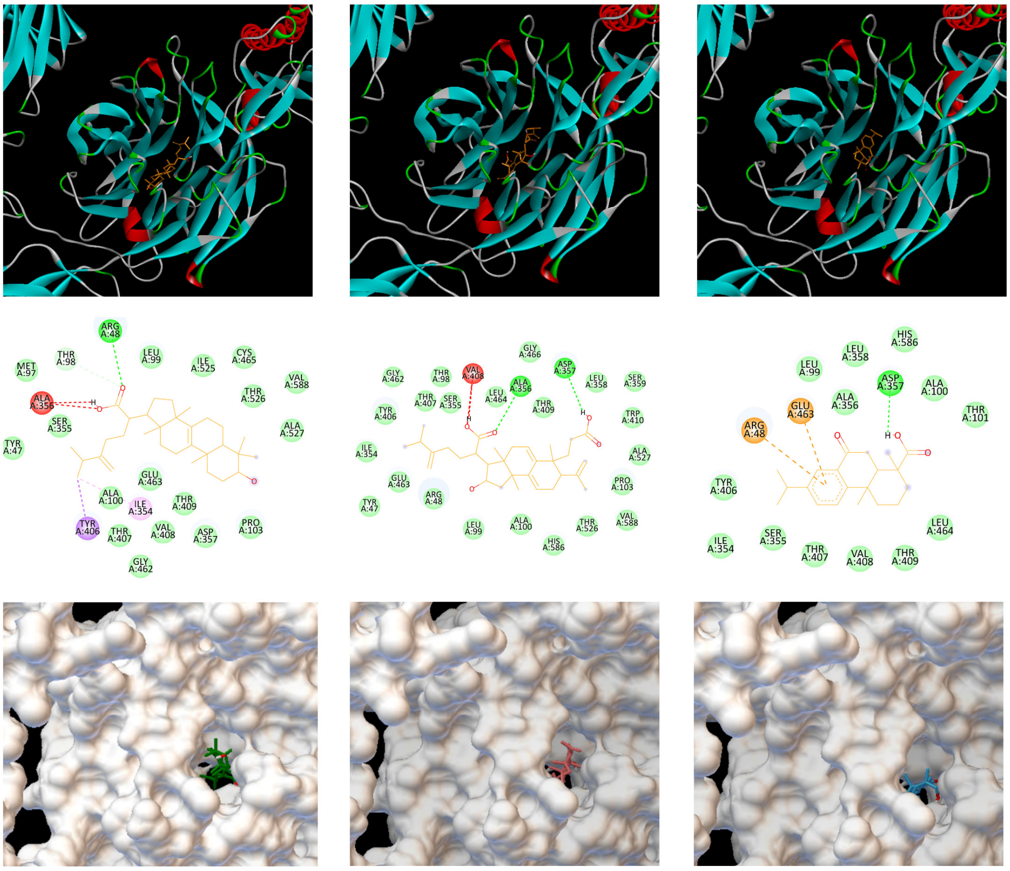

Figure S1. Docking results.

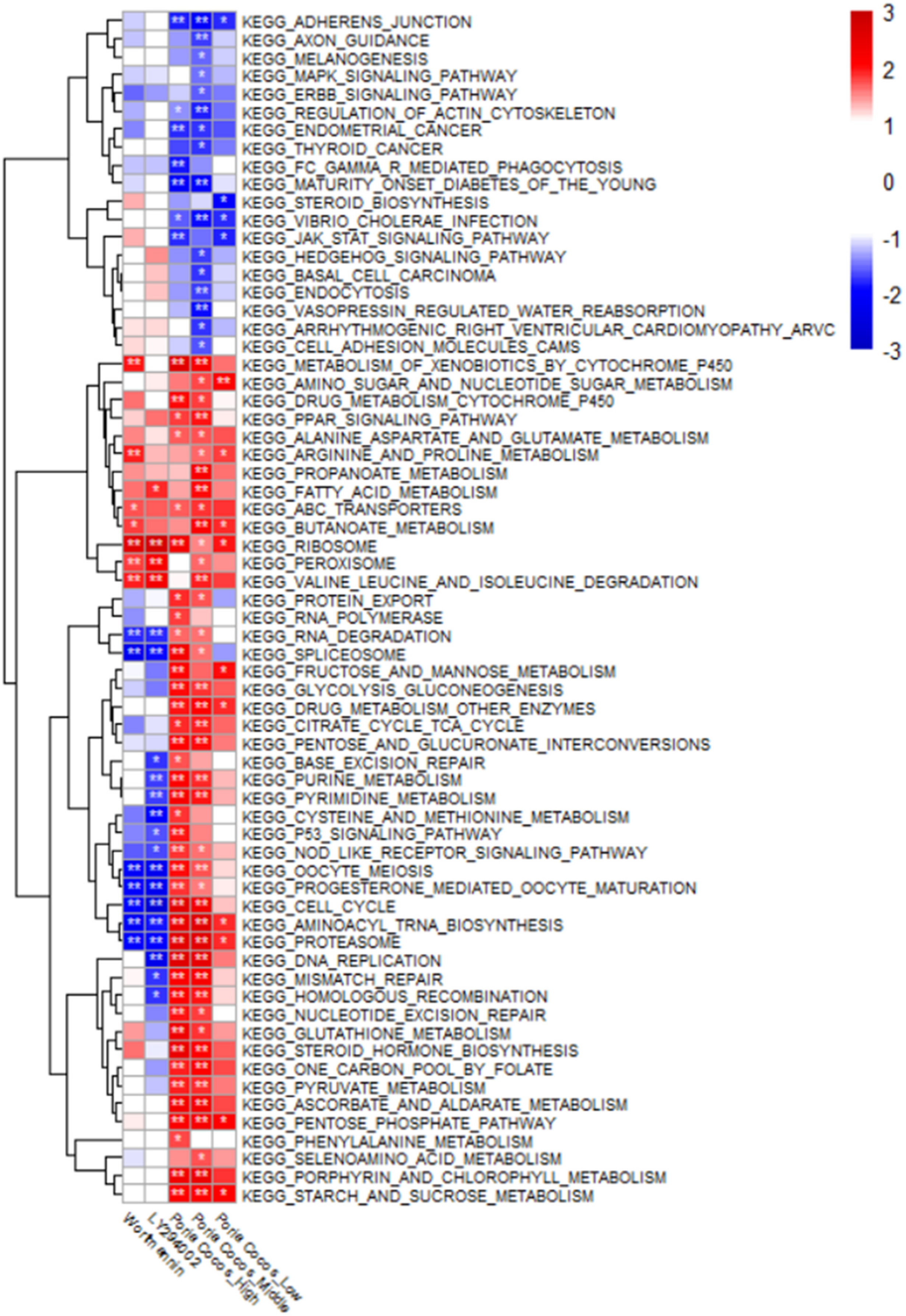

Figure S2. GSEA results using the HT29 cell line-based PC-induced transcriptomes. * $p < 0.05$, ** $p < 0.01$.

(A) Wnt signaling pathway & Zn to anterograde axonal transport

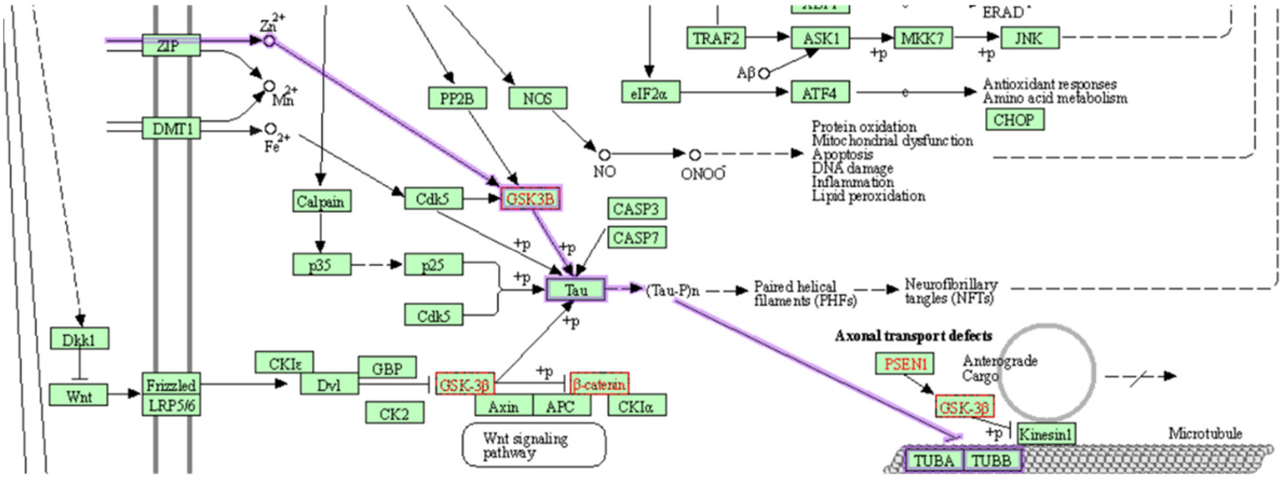

(B) Impairment of autophagy

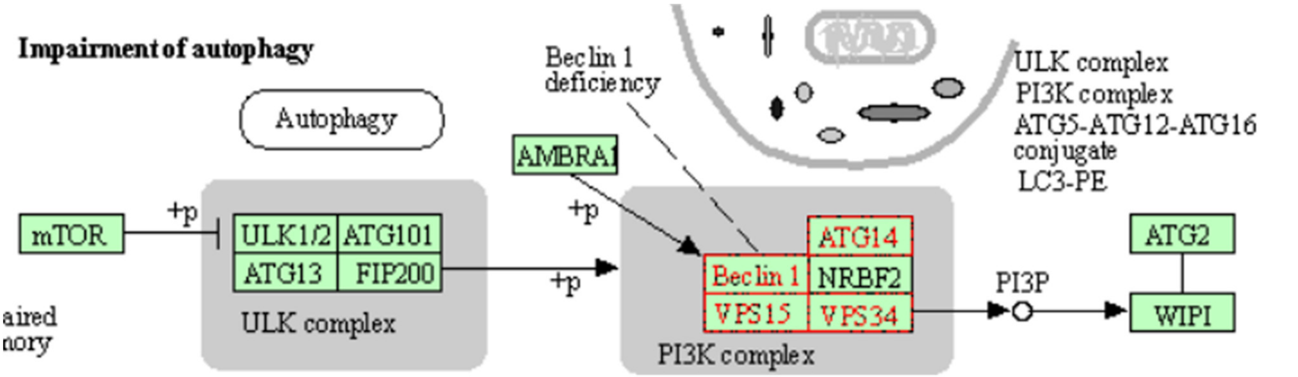

(C) Insulin signaling pathway

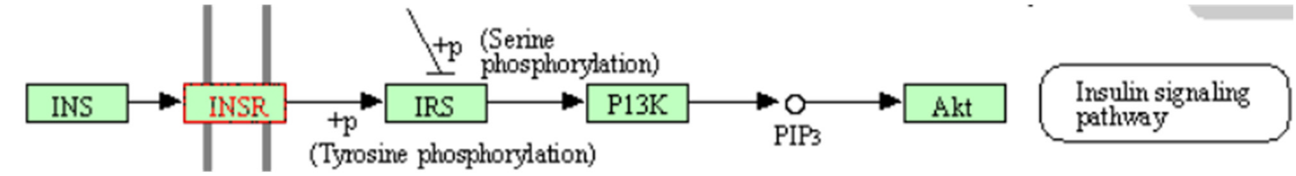

(D) AGE-RAGE signaling pathway (Mutation-caused aberrant Abeta to AGE-RAGE signaling pathway)

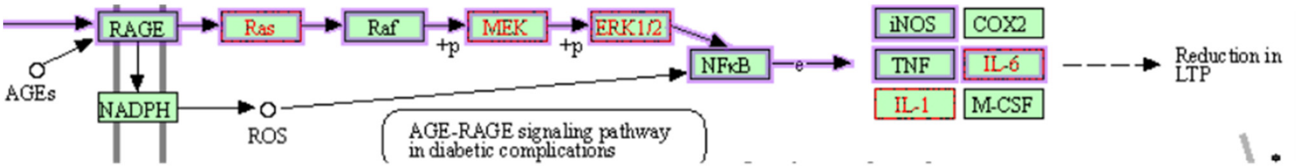

(E) Apoptosis (Mutation-caused aberrant Abeta to crosstalk between extrinsic and intrinsic apoptotic pathways)

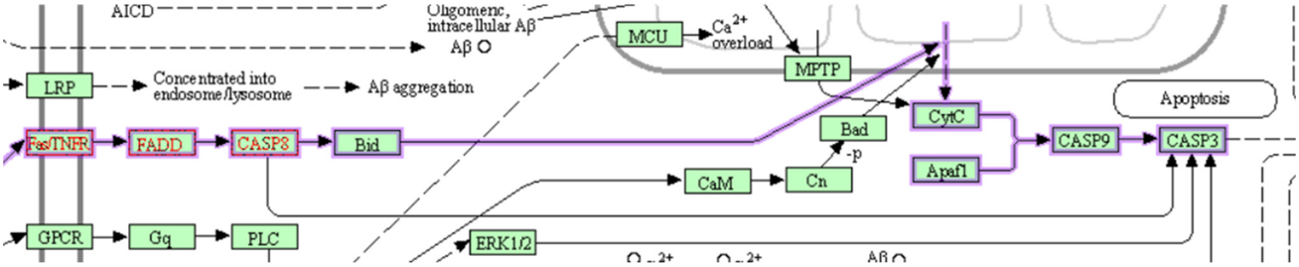

Figure S3. Protein signaling pathway identified in the kegg pathway database.
