# Supplementary material for: Effect of Poria cocos Terpenes: Verifying Modes of Action Using Molecular Docking, Drug-Induced Transcriptomes, and Diffusion Network Analyses
Source: Int J Mol Sci. 2024 Apr 24;25(9):4636. doi: 10.3390/ijms25094636 (PMC11083729; doi:10.3390/ijms25094636)
Supplement: Supplementary file 1 [file ijms-25-04636-s001.zip › Supplementary Table S1. Terpenes in Poria cocos.pdf]

Supplementary Table S1. Terpenes in *Poria cocos.*

Terpene Name	Dockability
**Lanostane-type triterpenes**	
Eburicoic acid	True
O-Acetylpachymic acid-25-ol	False
3*β*-Hydroxy-16*α*-acetoxy-lanosta-7,9(11),24-trien-21-oic acid	False
Pachymic acid	True
Lanostane	True
Polyporenic acid C	True
3-epi-Dehydrotumulosic acid	True
25-Hydroxy-3-epidehydrotumulosic acid	True
Versisponic acid E	True
3β,16α-Dihydroxylanosta-7,9(11),24-trien-21-oic acid	True
Dehydroeburiconic acid	True
Daedaleanic acid B	True
16α-Hydroxyeburiconic acid	True
3-epi-Dehydrotrametenolic acid	True
Trametenolic acid	True
Tumulosic acid	True
Dehydrotumulosic acid	True
3-O-Acetyl-16α-hydroxytrametenolic acid	True
3-O-Acetyl-16α-hydroxydehydrotrametenolic acid	True
3-epi-Dehydropachymic acid	True
Dehydropachymic acid	True
Dehydroeburicoic acid	True
Dehydroeburicoic acid monoacetate	True
3β-p-Hydroxybenzoyldehydrotumulosic acid	True
Dehydrotrametenolic acid	True
Pinicolic acid A	True

Masticadienoic acid	True
16α,27-Dihydroxydehydrotrametenoic acid	True
15α-Hydroxydehydrotumulosic acid	True
5α,8α-Peroxydehydrotumulosic acid	True
16α,25-Dihydroxydehydroeburicoic acid	True
Dehydrotrametenonic acid	True
25-Hydroxy-3-epitumulosic acid	True
Acetyleburicoic acid	True
16α,25-Dihydroxyeburiconic acid	True
29-Hydroxydehydrotumulosic acid	True
3α-acetoxylanosta-8,24-dien-21-oic acid	False
Ganoderic acid	True
6α-Hydroxypolyporenic acid C	True
Pinicolic acid E	True
Eburicoic acid acetate	True
16α-Hydroxytrametenolic acid	True
3*β*,16*α*,30-Trihydroxy-24-methyllanosta-7,9(11),24(31)-trien-21-oic acid	True
16*α*-Hydroxy-3-oxo-24-methyllanosta-5,7,9(11),24(31)-tetraen-21-oic acid	True
3*β*,16*α*-Dihydroxy-7-oxo-24-methyllanosta-8,24(31)-dien-21-oic acid	True
3*α*,16*α*-Dihydroxy-7-oxo-24-methyllanosta-8,24(31)-dien-21-oic acid	True
29-Hydroxydehydropachymic acid	True
3-(2-Hydroxyacetoxy)-5*α*,8*α*-peroxydehydrotumulosic acid	True
3*β*-Acetoxylanosta-7,9(11),24-trien-21-oic acid	True
29-Hydroxypolyporenic acid C	True
3-Epi-pachymic acid	True
6,9-Epoxyergosta-7,22-diene-3-ol	True
3*β*-Acetoxy-16*α*,24*β*-dihydroxylanosta-7,9(11),25-trien-21-oic acid	True
Masticadienolic acid	False
Daedaleanic acid F	True

Porilactone A	True
Porilactone B	True
Pinicolic acid F	True
Ceanphytamic acid A	True
Ceanphytamic acid B	True
15α-Hydroxy-3-oxolanosta-8,24-dien-21-oic acid	False
16,27-dihydroxyl-dehydrotrametenolic acid	False
16-O-acetylpachymic acid	False
16α-acetoxy-26,27-dimethoxyl-lanosta-8,24(31)-dien-21-oic acid	False
16α-Acetoxy-lanosta-8,24-dien-21-oic acid	False
16α-Acetoxypolyporenic acid C	False
16α-acetyloxy-24-methylene-3-oxolanosta-7,9(11)-dien-21-oic acid	False
16α-Acetyloxyeburiconic acid	False
16α-Hydroxy-3-oxolanosta-7,9(11),24-trien-21-oic acid	False
16α-Hydroxy-3-oxolanosta-8,24-dien-21-oic acid	False
16α-Hydroxydehydropachymic acid	False
16α-Hydroxy-lanosta-7,9(11),24-trien-21-oic acid	False
16α-Hydroxy-lanosta-8,24(31)-dien-21-oic acid	False
16α-Hydroxy-lanosta-8,24-dien-21-oic acid	False
25-Hydroxy-3-epi-hydroxytumulosic acid	False
25-Hydroxypachymic acid	False
25-Hydroxypolyporenic acid C	False
25α-Hydroxytumulosic acid	False
3,15-O-diacetyl-dehydrotrametenolic acid	False
3,24-Dioxo-16α-hydroxylanosta-7,9(11)-dien-21-oic acid	False
31-Hydyoxyl-16-O-acetylpachymic acid	False
3-Acetyloxy-16α-hydroxytrametenolic acid	False
3-epi-(3'-hydroxy-3'-methylglutaryloxyl)-16α-hydroxydehydrotrametenolic acid	False

3-epi-(3'-hydroxyl-3'-methylglutaryloxyl)-dehydrotumulosic acid	False
3-epi-(3'-hydroxyl-3'-methylglutaryloxyl)-tumulosic acid	False
3-epi-(3'-O-methyl malonyloxy)-dehydrotumulosic acid	False
3-O-Acetyl-16α,26-dihydroxytrametenolic acid	False
3-oxo-16α,25-Dihydroxylanosta-7,9(11),24(31)-trien-21-oic acid	False
3-oxo-16α-Hydroxylanosta-7,9(11),24(31)-trien-21-oic acid	False
3-oxo-16α-Hydroxylanosta-7,9(11),24-trien-21-oic acid	False
3-oxo-6,16α-Dihydroxylanosta-7,9(11),24(31)-trien-21-oic acid	False
3-Oxo-6,16α-dihydroxytrametenolic acid	False
3α,16β-Dihydroxylanosta-7,9(11),24-trien-21-oic acid	False
3β,5α,9α-Trihydroxy-ergosta-7,22-diene-6-one	False
3β-Acetoxyl-16α-hydroxy-lanosta-8,24(31)-diene-21-oic acid	False
3β-Acetyloxy-16α-hydroxylanosta-7,9(11),24(31)-trien-21-oic acid	False
3β-Acetyloxy-16α-hydroxylanosta-7,9(11),24(31)-trien-21-oic acid	False
6α-hydroxydehydropachymic acid	False
Acetoxyeburicoic acid	False
Coriacoic acid A	False
Coriacoic acid B	False
Coriacoic acid C	False
Coriacoic acid D	False
Dehydrosulphurenic acid	False
Ergosta-4,22-diene-3-one	False
Ergosta-5,6-epoxy-7,22-dien-3-ol	False
Hispindic acid B	False
Lanosta-7,9(11),24(31)-trien-21-oic acid	False
Lanosta-7,9(11),24-trien-21-oic acid	False
Lanosta-8,24(31)-dien-21-oic acid	False
Lanosta-8,24-dien-21-oic acid	False
Methyl trametenolate	False

Methyl-O-acetylpachymate	False
O-Acetylpachymic acid	False
Oxotrametenolic acid	False
Pachymic acid methyl ester	False
Pinicolic acid	False
Poriacosone A	False
Poriacosone B	False
Poricoic acid ZF	False
Poricoic acid ZH	False
Poricoic acid ZI	False
Poricoic acid ZL	False
**Seco-Lanostane-type triterpenes**	
Poricoic acid A	True
Poricoic acid B	True
Poricoic acid G	True
Poricoic acid H	True
Daedaleanic acid A	True
Poricoic acid E	True
Poricoic acid BM	True
Poricoic acid CM	True
16-Deoxyporicoic acid B	True
25-Hydroxyporicoic acid H	True
Poricoic acid D	True
26-Hydroxyporicoic acid DM	True
25-Hydroxyporicoic acid C	True
6,7-Dehydroporicoic acid H	True
Poricoic acid AM	True
Poricoic acid DM	True
Poricoic acid GM	True

Poricoic acid HM	True
Poricotriol A	True
Poricoic acid C	True
Poricoic acid AE	True
Poricoic acid CE	True
3-o-acetyl-dehydroeburicoic acid	True
Daedaleanic acid D	True
Poricoic acid I	True
Poricoic acid J	True
Poricoic acid K	True
Poricoic acid L	True
Poricoic acid M	True
Daedaleanic acid E	True
11β-Ethoxydaedaleanic acid A	True
16-Deoxyporicoic acid BM	False
25-Methoxy-29-hydroxyporicoic acid	False
26-Hydroxyporicoic acid G	False
3,4-Secolanosta-4(28),7,9,24Z-tetraen-3,26-dioic acid	False
Poricoic acid GE	False
Poricoic acid HE	False
Poricoic acid JM	False
Poricoic acid N	False
Poricoic acid O	False
Poricoic acid ZA	False
Poricoic acid ZC	False
Poricoic acid ZD	False
Poricoic acid ZG	False
Poricoic acid ZK	False
Poricoic acid ZM	False

Poricoic acid ZN	False
Poricoic acid ZO	False
Poricoic acid ZP	False
Poricoic acid ZQ	False
Poricoic acid ZR	False
**Pentacyclic triterpenes**	
Oleanolic acid	True
Hederagenin	True
β-Amyrin acetate	True
3-O-Acetyloleanolic acid	True
Oleanic acid 3-O-acetate	False
Triterpene acid	False
**Tricyclic diterpenes**	
1,7-Oxocallitrisic acid	True
Dehydroabietic acid	True
Pimaric acid	True
Dehydroabietic acid methyl ester	True
7-oxo-15-Hydroxydehydroabietic acid	True
**Sterols**	
Ergosta-7,22-dien-3-one	True
β-Sitosterol	True
Ergosterol	True
ergosta-7,22E-dien-3beta-ol	True
Ergosta-5,7-dien-3β-ol	True
Ergosterol peroxide	True
Daucosterol	True
(22E)-Ergosta-5,7,9(11),22-tetraen-3β-ol	True
Biemnasterol	True
Cerevisterol	True

Ergosterone	True
9,11-Dehydroergosterol peroxide	True
Ergost-7-en-3β-ol	True
Ergosta-4,22-dien-3-one	True
3β,5α-Dihydroxy-ergosta-7,22-dien-6-one	True
6,9-Epoxy-ergosta-7,22-dien-3-ol	True
(22E)-Ergosta-6,8(14),22-trien-3β-ol	False
(22E)-Ergosta-8(14),22-dien-3β-ol	False
(22E,24R)-ergosta-7,22-dien-3-one	False
3β,5α,9α-Trihydroxy-ergosta-7,-dien-6-one	False
**Other compounds**	
Caprylic acid	True
Palmitic acid	True
Lauric acid	True
Mannitol	True
Ribitol	True
Undekansaeure	True
Protocatechualdehyde	True
(-)-Pinoresinol	True
Trimethyl citrate	True
Ethyl glucoside	True
2-Lauroleic acid	True
Dimethyl L-malate	True
L-uridine	True
6-Hydroxykaempferol 7-glucopyranoside	False
Poricoic acid ZG	False
